# Civil Service Examination for Physicians

**Published:** 1891-08

**Authors:** 


### CIVIL SERVICE EXAMINATION FOR PHYSICIANS.

The advantages to the public at large of appointment to the civil service by competitive examination were never better illustrated than in the examination of physicians for the places of district physicians and sanitary inspectors, lately held in this city. According to the report made to the commission at its July meeting, there were twenty-two applicants, of whom only seven—less than one-third—succeeded in passing. The examination was not a difficult one, and contained only such questions as a well-informed physician should be able to answer. The fact that, of those who succeeded in passing, all but one obtained a high average, proves that the questions were not unfair.

If the same proportion holds good among the rest of the physicians of the city, it is a sad thing for the citizens to reflect upon, that more than two-thirds of our physicians are really unfit for their calling. Such revelations as this show how great need there is for the strict carrying out of the provisions of the new law providing for State examination for license to practice medicine.

